# Parental Stress, Depression, and Participation in Care Before and During the COVID-19 Pandemic: A Prospective Observational Study in an Italian Neonatal Intensive Care Unit

**DOI:** 10.3389/fped.2021.737089

**Published:** 2021-09-30

**Authors:** Jenny Bua, Ilaria Mariani, Martina Girardelli, Murphy Tomadin, Antonella Tripani, Laura Travan, Marzia Lazzerini

**Affiliations:** ^1^Neonatal Intensive Care Unit, Institute for Maternal and Child Health IRCCS “Burlo Garofolo”, Trieste, Italy; ^2^WHO Collaborating Centre for Maternal and Child Health, Institute for Maternal and Child Health IRCCS “Burlo Garofolo”, Trieste, Italy; ^3^Department of Pediatrics, Institute for Maternal and Child Health IRCCS “Burlo Garofolo”, Trieste, Italy

**Keywords:** parents, stress, depression, participation, COVID-19, neonatal ICU

## Abstract

**Background:** Recent studies reported, during the COVID-19 pandemic, increased mental distress among the general population and among women around the childbirth period. COVID-19 pandemic may undermine the vulnerable well-being of parents in Neonatal Intensive Care Units (NICUs).

**Objective:** Our study aimed to explore whether parental stress, depression, and participation in care in an Italian NICU changed significantly over three periods: pre-pandemic (T_0_), low (T_1_), and high COVID-19 incidence (T_2_).

**Methods:** Enrolled parents were assessed with the Parental Stressor Scale in the NICU (PSS:NICU), Edinburgh Postnatal Depression Scale (EPDS), and Index of Parental Participation (IPP). Stress was the study primary outcome. A sample of 108 parents, 34 for each time period, was estimated to be adequate to detect a difference in PSS:NICU stress occurrence level score (SOL) of 1.25 points between time periods. To estimate score differences among the three study periods a non-parametric analysis was performed. Correlation among scores was assessed with Spearman rank coefficient.

**Results:** Overall, 152 parents were included in the study (62 in T_0_, 56 in T_1_, and 34 in T_2_). No significant differences in the median PSS:NICU, EPDS, and IPP scores were observed over the three periods, except for a slight increase in the PSS:NICU parental role sub-score in T_2_ (T_0_ 3.3 [2.3–4.1] vs. T_2_ 3.9 [3.1–4.3]; *p* = 0.038). In particular, the question regarding the separation from the infant resulted the most stressful aspect during T_2_ (T_0_ 4.0 [4.0–5.0] vs. T_2_ 5.0 [4.0–5.0], *p* = 0.008). The correlation between participation and stress scores (*r* = 0.19–022), and between participation and depression scores (*r* = 0.27) were weak, while among depression and stress, a moderate positive correlation was found (*r* = 0.45–0.48).

**Conclusions:** This study suggests that parental stress and depression may be contained during the COVID-19 pandemic, while participation may be ensured.

## Introduction

Research has indicated that, in ordinary times, the experience of a neonatal intensive care unit (NICU) hospitalization is a distressing and potentially traumatic event for parents (1). During the current COVID-19 pandemic, multiple studies reported increased stress, anxiety, and depression among the general population and women around childbirth (2, 3). Parents of newborns hospitalized in the NICU may be particularly exposed to stressors associated with COVID-19, such as increased worries about newborn health, need for physical distancing, restrictions in NICU visitation, overall reorganization of health services, and reduced social and family support (1–3). Family-centered care is essential to mitigating mental distress of NICU parents and improving the health and well-being of their infants, and several authors have called for monitoring and support of NICU parents' well-being during the COVID-19 pandemic (4, 5).

So far, no study has explored how the COVID-19 pandemic affected parental stress, depression, and participation in care of their neonates admitted to the NICU. This study aimed at exploring whether, in an Italian NICU, parental stress, depression, and participation in care changed significantly over three time periods: before the pandemic and during periods of low and high COVID-19 incidence. Secondary aim was to assess the correlation between parental stress, depression and participation. This preliminary study is the first publication of the baseline assessment of an ongoing multicentre project called “Empowering Parents in the NICU” (EPINICU) exploring models to increase participation in NICU care in four different settings (Italy, Tanzania, Brazil, Sri Lanka). The EPINICU study was approved by the Ethical Committee of Friuli Venezia Giulia Region, Italy (Prot.31633, October 22, 2019).

## Materials and Methods

### Study Design, Study Setting, and Participants

This was a descriptive cross-sectional study, and the Strengthening the Reporting of Observational Studies (STROBE) in Epidemiology guidelines were applied (6).

It was conducted in the NICU of a referral pediatric hospital in the North-East of Italy. Since the 1980s, our NICU has been a 24/7 open ward for both parents. It is a small Open Bay NICU with 24 cots (10 in intensive care, 12 in semi-intensive care divided in three rooms, two in an isolated negative-pressure room). In the last 2 years, it handled an average of 260 NICU admissions/year. During the whole study period, a total of nine neonatologists, 24 nurses, two unit psychologists were in service.

Mothers and fathers of newborns who have been hospitalized in the NICU or semi-intensive care for at least 48 h, with an age of at least 18 years, and fluent in Italian were enrolled prospectively. Parents with previously diagnosed mental disorders, parents of newborns dead at birth or during hospitalization, not fluent in Italian or not providing consent were excluded.

All participants provided a written consent before responding to questionnaires.

### Data Collection Procedures

Data were collected over three time periods: pre-pandemic (26th November 2019–1st March 2020, T_0_); summertime just after the first Italian wave characterized by low incidence of COVID-19 cases (1st May−31st August 2020, T_1_); autumn with high COVID-19 incidence (1st September−30th November 2020, T_2_) as documented by the official statics from the Italian National Institute of Health (7). The study was temporarily interrupted at the very beginning of the pandemic and of the Italian lockdown (i.e., between 2nd March and 30th April) as the hospital priority was to react to the emergency and organize specific COVID-19 dedicated paths both for newborns, children, and pregnant women, while most research activities were suspended.

The study and questionnaires were introduced to parents either by a neonatologist or NICU psychologists close to discharge, with a median of 5 days before discharge (IQR 1–10). Three self-administrated questionnaires were used. Stress was measured with the Parental Stressor Scale (PSS) for NICU (8), validated in Italy (9) and including 26 statements divided in three sections: stress due to Sights and Sounds (6 items), to Infant Behavior and Appearance (13 items) and to Parental Role Alteration (7 items). PSS:NICU includes also a final question about overall stress during NICU hospitalization. Answers for each question are on a Likert scale from 1 (“no stress”) to 5 (“extremely stressful”).

Parental depression was measured with the Edinburgh Postnatal Depression Scale (EPDS), which has been validated for use in several countries, including Italy (10). The scale includes 10 questions with four possible answers each, and a total score ranging from 0 to 30.

Parental participation was measured with the Index of Parental Participation (IPP), modified to describe activities related to newborn care. The adaptation was approved and back translated by Dr. Melnik, the author of the instrument (11). The IPP includes 30 dichotomous (yes/no) items, divided in four sections: activities related to Daily Living (6 items); Providing Comfort (7 items); Advocating for newborn health (7 items); Technical Tasks (10 items). The total score ranges from 0 to 30, with higher scores indicating higher parental participation in care.

### Data Analysis

Stress was the study primary outcome. A sample of 108 parents, 34 for each time period, was estimated to be adequate to detect a difference in PSS:NICU stress occurrence level score (SOL; see below for definition) of 1.25 points between time periods, deemed to be clinically relevant and assuming an overall type I error of 0.05, 99% power and a conservative standard deviation of 1 (8) and using a Wilcoxon-Mann Whitney test.

PSS scores were calculated according to Miles et al. (8), using two methods: (a) the Stress Occurrence Level (SOL) calculated including only experienced items; (b) the Overall Stress Level (OSL) scoring “not applicable items” with one point. These two methods provide complementary information and have the following justification in literature: SOL should be used when the focus is the parent as it captures real lived experience, while OSL when the focus is the NICU environment (8).

An EPDS score ≥12 was used as a cut-off for depression screening, since literature showed that in the female and male Italian population, this is the most appropriate diagnostic cut-off (10, 12).

Summary statistics are presented as absolute frequencies and percentages, and as medians and interquartile ranges (IQR) for continuous non-normally distributed data. Due to deviation from parametric model assumptions, to estimate score differences among periods a non-parametric analysis was performed using the Kruskal-Wallis test. Bonferroni adjusted *p*-values were calculated for multiple comparisons among time periods tested with the Wilcoxon-Mann Whitney. A subgroup analysis by periods was performed to evaluate whether parental outcomes differed according to unit of admission of their newborn (NICU vs. semi-intensive) using a Wilcoxon-Mann Whitney test. Correlation between scores was analyzed using Spearman rank correlation coefficients. All tests performed were two tailed and a *p*-value of <0.05 was considered statistically significant. Statistical analyses were performed using Stata version 14 and R version 3.6.1.

## Results

Of the 259 newborns admitted to our Neonatal Unit during the study period (26/11/2019–30/11/2020), 96 (37%) were excluded as they did not meet the inclusion criteria. Parents of 64 (25%) newborns refused to participate (3/64) or were not approached for enrolment (61/64). Overall, we included 152 parents (91 mothers, 61 fathers) (Supplementary Figure 1). Newborns of included parents (*N* = 99) did not differ from those whose parents were not enrolled (*N* = 64) except for having a lower birthweight (2450.0 [1890.0, 3260.0] vs. 2880.0 [2385.0, 3535.0] g, *p* = 0.004), a longer length of stay (14.0 [8.0, 23.5] vs. 5.0 days [4.0, 11.0], *p* < 0.001) and higher frequency of ventilation at birth (21.2 vs. 4.7%, *p* = 0.003) (Supplementary Table 1).

Parental (Table 1) and newborn (Supplementary Table 2) characteristics did not differ significantly between the parents' groups in the three study periods, except for a higher percentage of female babies (T_0_ 50%, T_1_ 46.2%, T_2_ 80%, *p* = 0.034), and of infants admitted to the NICU compared to the semi-intensive unit in T_2_ (T_0_ 15%, T_1_ 46.2%, T_2_ 60%, *p* = 0.001) (Supplementary Table 2).

**Table 1:** Parents' characteristics during three COVID-19 pandemic periods.

	**Overall**	**Pre-pandemic (T_**0**_)**	**Low COVID-19 incidence (T_**1**_)**	**High COVID-19 incidence (T_**2**_)**	***p*-value**
	***n* (%)**	***n* (%)**	***n* (%)**	***n* (%)**	
***N***	152	62	56	34	
**Age Median [IQR]**	35.0 [31.0, 39.0]	35.0 [32.0, 39.0]	35.0 [30.0, 40.0]	34.0 [29.8, 38.0]	0.581
**Role**					
Father	61 (40.1)	25 (40.3)	21 (37.5)	15 (44.1)	0.824
Mother	91 (59.9)	37 (59.7)	35 (62.5)	19 (55.9)	0.824
**Education**					
Lower secondary	24 (15.8)	11 (17.7)	8 (14.3)	5 (14.7)	0.845
Upper secondary	65 (42.8)	25 (40.3)	23 (41.1)	17 (50.0)	0.670
Higher	61 (40.1)	24 (38.7)	25 (44.6)	12 (35.3)	0.675
**Working status**					
Working	129 (84.9)	51 (82.3)	48 (85.7)	30 (88.2)	0.709
Un-employed	19 (12.5)	9 (14.5)	7 (12.5)	3 (8.8)	0.737
Missing	4 (2.6)	2 (3.2)	1 (1.8)	1 (2.9)	1.000
**Marital status**					
Married	67 (44.1)	27 (43.5)	29 (51.8)	11 (32.4)	0.197
Un-married	85 (55.9)	35 (56.5)	27 (48.2)	23 (67.6)	0.197
**Parity (recorded only for mothers)**					
1	50 (32.9)	21 (33.9)	18 (32.1)	11 (32.4)	0.924
2	26 (17.1)	13 (21.0)	10 (17.9)	3 (8.8)	0.330
>2	14 (9.2)	3 (4.8)	6 (10.7)	5 (14.7)	0.167
Missing	1 (0.7)	0 (0.0)	1 (1.8)	0 (0.0)	0.593

**Figure 1:**
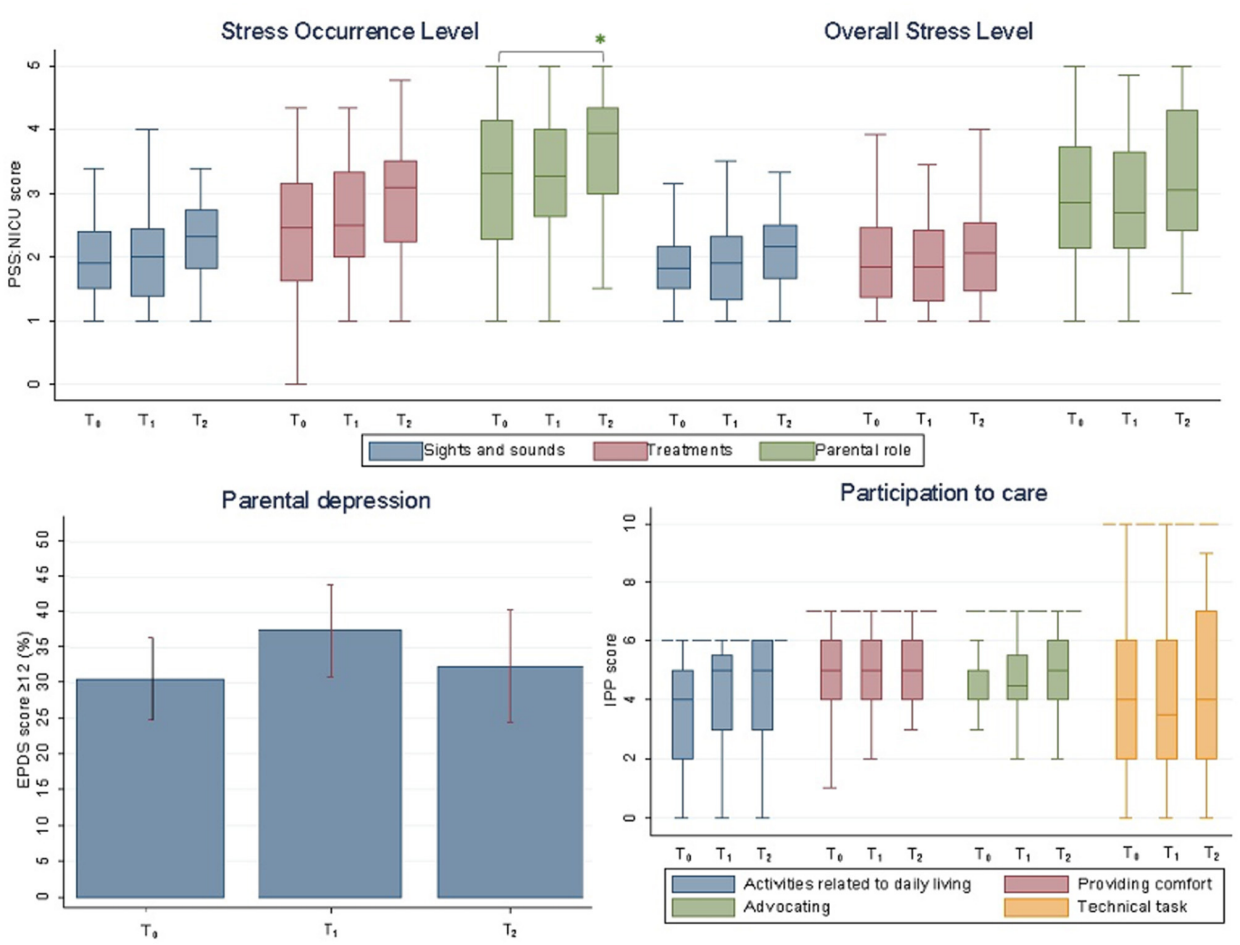
Stress (PSS:NICU), depression (EPDS), and participation in care (IPP) during three COVID-19 pandemic periods. PSS:NICU (Stress Occurrence Level and Overall Stress Level) and IPP scores are presented as medians. EPDS ≥12 is presented as percentage and standard deviation. Maximum scores vary for each IPP subdomain and are graphically shown with a dashed line. *statistically significance; T_0_, pre-pandemic; T_1_, low COVID-19 incidence; T_2_, high COVID-19 incidence. PSS:NICU, Parenteral Stressor Scale in the NICU; EPDS, the Edinburgh Postnatal Depression Scale; IPP, Index of Parental Participation.

Overall, moderate to high levels of stress (median PSS:NICU=3 [3.0–4.0]) were observed in our population, with 34% of parents scoring ≥12 at EPDS (Table 2). No significant differences in the percentage of EPDS ≥12 and in median PSS:NICU and IPP scores were observed in the three periods (Figure 1), with the exception of a slight increase in the PSS:NICU parental role sub-score in T_2_, when the score was calculated as SOL (T_0_ 3.3 [2.3–4.1] vs. T_2_ 3.9 [3.1–4.3], adjusted *p* = 0.038) (Table 2). Difference in median PSS:NICU score was due to a single question measuring stress related to separation from the newborn (T_0_ 4.0 [4.0–5.0] vs. T_2_ 5.0 [4.0–5.0], *p* = 0.008) (Supplementary Table 3).

**Table 2:** PSS-NICU, EPDS, and IPP median scores in three COVID-19 pandemic periods.

	**Overall**	**Pre-pandemic (T_**0**_)**	**Low COVID-19 incidence (T_**1**_)**	**High COVID-19 incidence (T_**2**_)**	***p-*value**	**Multiple comparisons** ***Adjusted p-value***		
	***Median [IQR]***	***Median [IQR]***	***Median [IQR]***	***Median [IQR]***		**T_**0**_-T_**1**_**	**T_**0**_-T_**2**_**	**T_**1**_-T_**2**_**
**PSS: NICU**	*N* = 152	*N* = 62	*N* = 56	*N* = 34				
**SOL**								
Sights and sounds	2.0 [1.5, 2.6]	1.9 [1.5, 2.4]	2.0 [1.4, 2.4]	2.3 [1.8, 2.7]	0.083	1.00	0.132	0.147
Treatments	2.6 [1.9, 3.4]	2.5 [1.6, 3.2]	2.5 [2.0, 3.3]	3.1 [2.3, 3.5]	0.142	0.818	0.201	0.692
Parental role	3.5 [2.6, 4.1]	3.3 [2.3, 4.1]	3.3 [2.7, 4.0]	3.9 [3.1, 4.3]	**0.028**	1.00	**0.038**	0.083
Total score	8.2 [6.4, 9.8]	7.5 [5.6, 9.3]	7.7 [6.5, 9.3]	9.4 [7.3, 10.2]	**0.031**	1.00	**0.037**	0.100
**OSL**								
Sights and sounds	1.9 [1.5, 2.3]	1.8 [1.5, 2.2]	1.9 [1.3, 2.3]	2.2 [1.7, 2.5]	0.132	1.00	0.202	0.232
Treatments	1.9 [1.4, 2.5]	1.8 [1.4, 2.4]	1.8 [1.3, 2.4]	2.1 [1.5, 2.5]	0.467	1.00	1.00	0.720
Parental role	2.9 [2.1, 3.7]	2.9 [2.1, 3.7]	2.7 [2.1, 3.6]	3.1 [2.5, 4.2]	0.141	1.00	0.241	0.210
Total score	6.9 [5.4, 8.5]	6.7 [5.2, 8.2]	6.6 [5.2, 8.3]	7.2 [5.9, 8.8]	0.165	1.00	0.357	0.185
**Final PSS:NICU question on overall stress**	3.0 [3.0, 4.0]	3.0 [2.2, 4.0]	3.0 [3.0, 4.0]	3.0 [2.2, 4.0]	0.831	1.00	1.00	1.00
**EPDS**	*N* = 150	*N* = 60	*N* = 56	*N* = 34				
Total score	9.0 [5.2, 13.0]	8.0 [4.8, 13.0]	8.5 [6.0, 13.2]	9.0 [6.0, 12.8]	0.615	1.00	1.00	1.00
score ≥12, *n* (%)	51 (34.0)	19 (31.7)	21 (37.5)	11 (32.4)	0.782	1.00	1.00	1.00
**IPP**	*N* = 152	*N* = 62	*N* = 56	*N* = 34				
Activities related to daily living	4.0 [3.0, 5.2]	4.0 [2.0, 5.0]	5.0 [3.0, 5.2]	5.0 [3.0, 5.8]	0.421	0.616	1.00	1.00
Providing comfort	5.0 [4.0, 6.0]	5.0 [4.0, 6.0]	5.0 [4.0, 6.0]	5.0 [4.0, 5.8]	0.631	1.00	1.00	1.00
Advocating	5.0 [4.0, 5.2]	5.0 [4.0, 5.0]	4.5 [4.0, 5.2]	5.0 [4.0, 6.0]	0.878	1.00	1.00	1.00
Technical task	4.0 [2.0, 6.0]	4.0 [2.0, 6.0]	3.5 [2.0, 6.0]	4.0 [2.0, 6.8]	0.806	1.00	1.00	1.00
Total score	18.0 [14.0, 21.2]	18.0 [15.0, 21.8]	18.0 [15.0, 21.2]	18.0 [13.2, 21.0]	0.957	1.00	1.00	1.00

*The bold values indicate the areas in which a p < 0.05 was observed*.

IPP and EPDS total scores did not differ between the three periods (Table 2). Increased parental participation in care was observed in T_2_ for two specific aspects regarding the change of clothes and diapers (Supplementary Table 4).

The subgroup analysis showed that in the three analyzed periods there were no significant differences in parental PSS:NICU, EPDS, and IPP scores according to unit of admission (data not shown).

**Figure 2:**
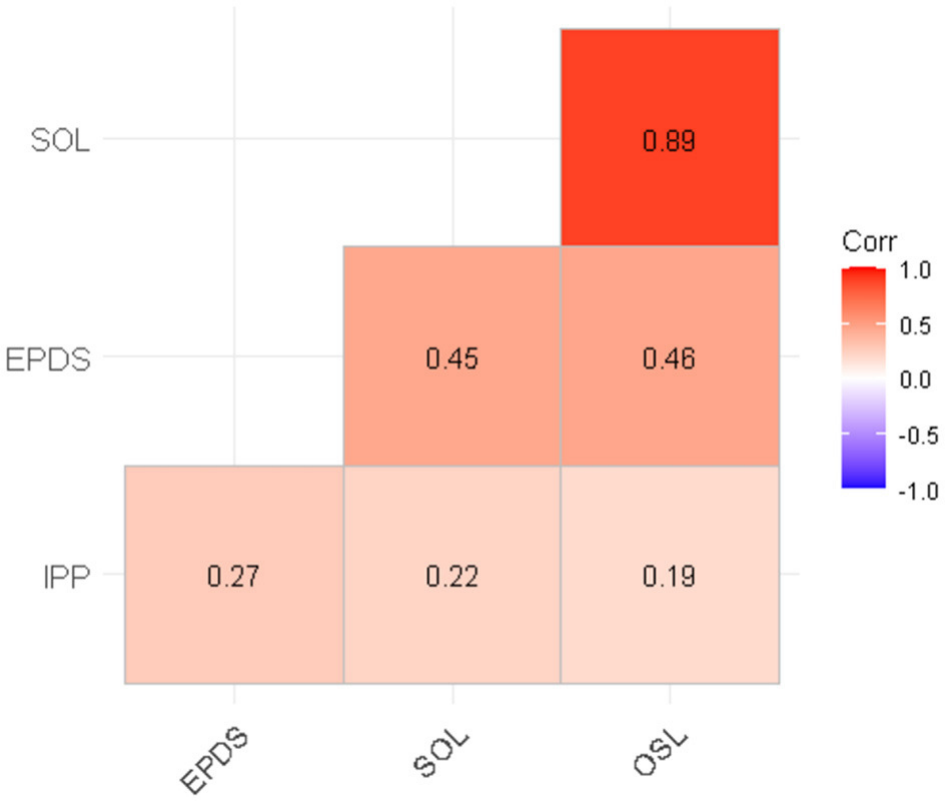
Correlation between IPP, EPDS and PSS:NICU SOL and OSL scores. All correlations were significant (*p* < 0.05). PSS:NICU, Parental Stressor Scale in the NICU; SOL, Stress Overall Level; OSL, Overall Stress Level; EPDS, the Edinburgh Postnatal Depression Scale; IPP, Index of Parenteral Participation.

The correlation between participation and stress score (*r* = 0.19–022), and between participation and depression scores (*r* = 0.27) were weak, while among depression and stress, a moderate positive correlation was found (*r* = 0.45–0.48) (Figure 2).

## Discussion

In our study, we found that parental stress, an EPDS value ≥12, and participation in care in an Italian NICU did not change significantly during the COVID-19 pandemic when compared with the period immediately before.

It is well-documented that NICU parents are at increased risk of stress and depression (1). Studies are difficult to compare as they use different cut-off values for the same mental health scale, they include different populations (mothers and/or fathers) and explore parental well-being at different times during hospitalization (13–15). Nonetheless, the rates of stress and depressive symptoms in our study resulted to be in line with the existent literature. A recent longitudinal study in parents of infants with lower gestational age at birth (<30 weeks) than our population showed that up to 40–50% had depressive symptoms, and, although rates decreased with time, they still remained above expected levels even after 12 weeks from admission (13). Alkozei reported that few days after NICU admission, mothers of pre-term babies reported rates of stress comparable to those reported by our parents (mean PSS:NICU stress score: 2.99 ± 0.85), while 38% of them had an EPDS ≥10 (14). As previously reported (14), we found that parental stress was consistently higher in the domain of the parental role (Figure 1). A recent meta-analysis on PSS:NICU confirmed that across different countries, the parental role is the greatest source of stress for both mothers and fathers in the NICU, while perceived stress seems to be independent from neonatal and clinical characteristics (15).

There are no data in the literature to compare our findings on parental participation in care in the NICU. According to the IPP scoring system, parental participation resulted to be medium-to-high in our study, and it was higher for activities related to daily living, comfort provision and advocacy rather than technical tasks, which in our NICU setting are usually carried out by nurses (Supplementary Table 4).

In our study we did not find changes in parental stress, depression, or participation in care during the COVID-19 pandemic. A lot of concern has been raised recently because the added burden of the COVID-19 pandemic may negatively impact the already vulnerable psychosocial health of NICU families (4, 5). A cross-sectional survey of 277 NICUs mostly from the USA highlighted profound restrictions in NICU visitation practices, such as a significant decrease in 24-h parental presence, and in parental participation in clinical rounds (16).

During the study period, visiting restrictions were applied but other organizational changes were enacted to mitigate the impact of these restrictions on families. Starting from T_1_, universal SARSCoV-2 testing with nasopharyngeal swabs and quantitative polymerase chain reaction tests was introduced for parents every 2 weeks and, since September 2020, every week. Despite visits were restricted to one parent per baby at the time, parents were allowed to enter as frequently and as long as they liked for a maximum of 2 per room in the semi-intensive unit. There was neither a decrease in the psychological support (two psychologists) nor in the medical and nursing staff. Psychologists were present in our NICU from Monday to Saturday and offered parental support also outside the unit by phone, as they did before COVID pandemic. During T_1_ and T_2_, all NICU staff had to wear a facial mask (surgical or FFP2), environmental cleaning procedures were intensified but no other major changes in care occurred. When parents were not present, daily updates on the baby's clinical condition were provided through video-calls by unit psychologists or the director. Recently telehealth has been advocated as a potential low-cost strategy in order to keep patients and providers safe, while emphasizing family-centered care (17).

All these aspects may have contributed in our NICU to ensure constant parental participation in care and stabilize stress and depressive symptoms, although our study was not designed as an intervention study to test this hypothesis.

We did observe higher SOL stress scores during T_2_, with separation from the baby deemed as the most stressful aspect. The higher percentage of babies in the NICU in T_2_ did not account for this difference. This result may well reflect a real difficulty related to the pandemic. As suggested by other authors (18, 19), the uncertainty feelings due to the pandemic may have boosted the emotional distress due to the separation from the baby. More so, as only one parent per baby at the time was allowed, the absence of the partner support during the NICU visits may have contributed to increase this feeling (18).

When exploring the correlation between scores, we found weak to moderate positive correlations between them. Evidence from RCTs showed that increased parental participation in care can reduce parental distress (20, 21). However, our study was not conceived as an interventional or longitudinal study. Data were collected cross-sectionally, hence we do not know whether and how participation affected stress/depression at different time points. It may have occurred that parents showing higher stress were invited to participate more in the care of their baby and in our study, we found that in T_2_, when Parental Role SOL was significantly higher, there was a significant increase in changing clothes and diapers when compared to T_0._ However, the IPP questionnaire may not capture some aspects of the quality of parental participation in care, although it contains several items on relationship with staff and advocacy (11). Finally, depression and self-perception of stress can have many other underlying causes, such as personality traits, history of depression and reported substance abuse (1) which were not investigated in our study.

Limitations of this study include that it was not designed to test any specific intervention, nor to look specifically at the associations between parental characteristics, participation in care, stress, and depression. Gender differences in stress and depression in NICU parents are reported (1, 15) but our sample size did not allow us to stratify data further. Sociodemographic characteristics of included parents could not be directly compared with those of not enrolled ones, as these data are not registered in the neonatal clinical records. However, newborns of included parents needed more intensive and longer clinical care than those of not enrolled parents, hence underestimating stress in our study population seems unlikely. We acknowledged the lack of data related to the start of the pandemic (i.e., 2nd March−30th April), however we believe this does not affect our findings related to T_2_, as it was a period characterized by a higher COVID-19 incidence.

Findings of our study cannot be directly generalized to other NICUs. More investigations on parental participation in care, in parallel with parental stress, anxiety and depression in different settings and over time shall further contribute to generate evidence on this often-neglected topic.

Further studies are also needed to evaluate specific interventions to mitigate the additional psychological burden of COVID-19, such as telematic approaches including telephone helplines, video calls, video tutorials and conferencing to see the hospitalized baby and to communicate with NICU staff (17, 19).

## Conclusion

Compared to other existing evidence, our study suggests that stress and depression levels of parents with infants admitted to NICU may remain unchanged even during peaks of the COVID-19 pandemic, and that parental participation in care can be maintained in these settings.

## Data Availability Statement

The raw data supporting the conclusions of this article will be made available by the authors, without undue reservation.

## Ethics Statement

The studies involving human participants were reviewed and approved by Institutional Review Board of Friuli Venezia Giulia Region. The patients/participants provided their written informed consent to participate in this study.

## Author Contributions

ML: conception of the EPINICU study. ML, JB, IM, and MG: conception of this paper. JB, MG, MT, and AT: collection of data. MT and MG: data entry. IM, JB, MG, and ML: analysis and interpretation of the data. JB and ML: drafting of the article. JB, MG, IM, AT, MT, LT, and ML: critical revision of the article for important intellectual content and final approval of the article. All authors contributed to the article and approved the submitted version.

## Funding

The study was supported by Chiesi Foundation research grant 2019 in Neonatology to our Institute of Research. As per the contract undersigned among parties, The Grant does not constitute, directly or indirectly, a fee for services provided or to be provided in favor of the Foundation. The funder had no role in study design, data collection and analysis, decision to publish, or preparation of the manuscript.

## Conflict of Interest

The authors declare that the research was conducted in the absence of any commercial or financial relationships that could be construed as a potential conflict of interest.

## Publisher's Note

All claims expressed in this article are solely those of the authors and do not necessarily represent those of their affiliated organizations, or those of the publisher, the editors and the reviewers. Any product that may be evaluated in this article, or claim that may be made by its manufacturer, is not guaranteed or endorsed by the publisher.
