# Supplementary material for: Parental Stress, Depression, and Participation in Care Before and During the COVID-19 Pandemic: A Prospective Observational Study in an Italian Neonatal Intensive Care Unit
Source: Front Pediatr. 2021 Sep 30;9:737089. doi: 10.3389/fped.2021.737089 (PMC8515023; doi:10.3389/fped.2021.737089)
Supplement: Supplementary file 1 [file Data_Sheet_1.docx]

Supplementary Material

Supplementary Figure 1. Flow chart of newborns enrolled in the study.


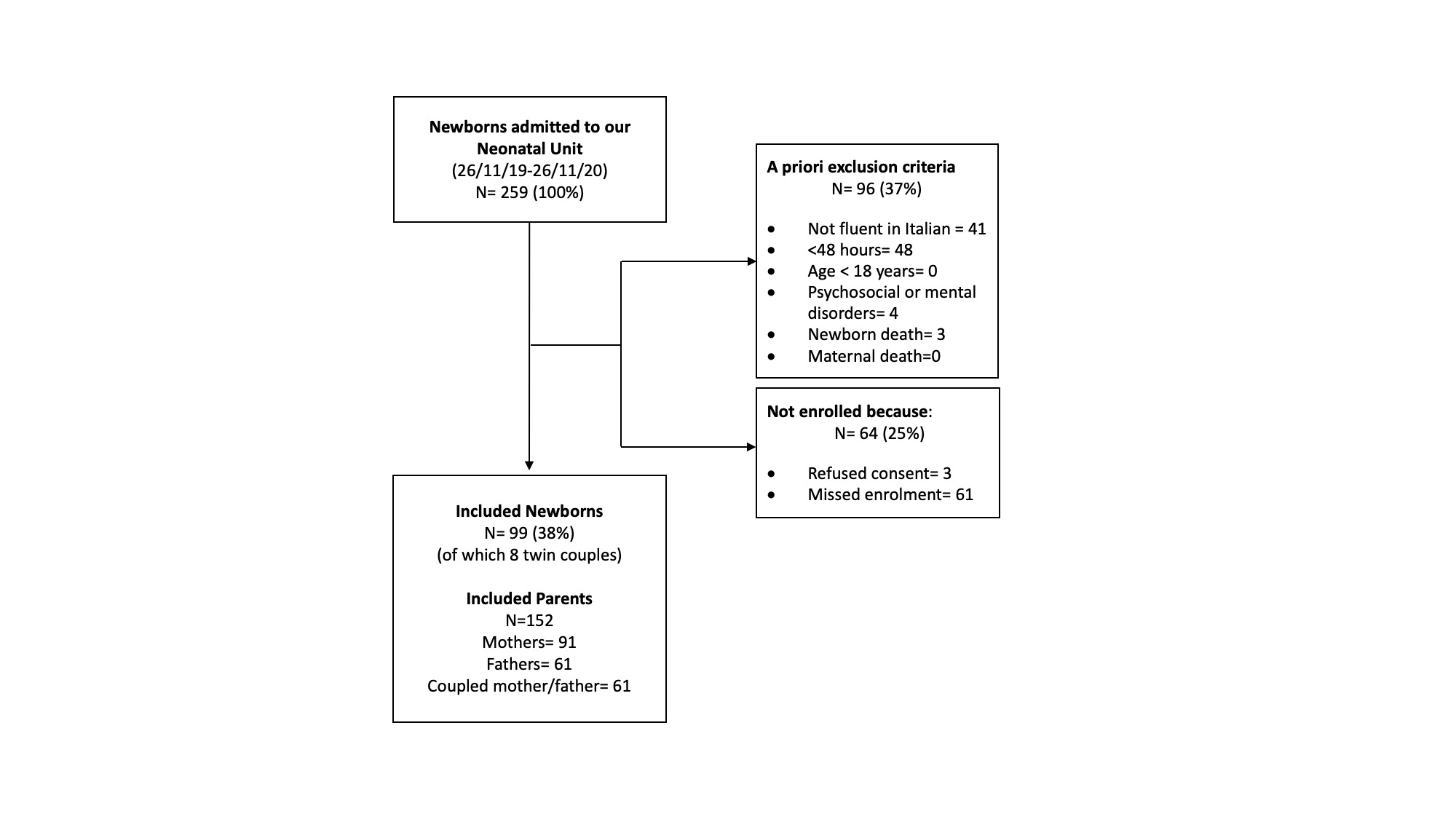


**Supplementary Table 1.** Comparison of newborns’ characteristics between newborns of enrolled parents and of not enrolled ones.

|  | **Enrolled**  ***n (%)*** | **Not enrolled**  ***n (%)*** | ***p-value*** |
| --- | --- | --- | --- |
| N | 99 | 64 |  |
| **Sex** |  |  | 0.057 |
| Female | 54 (54.5) | 25 (39.1) |  |
| Male | 45 (45.5) | 39 (60.9) |  |
| **Gestational age, weeks** |  |  | 0.055 |
| <=27 | 3 (3.0) | 1 (1.6) |  |
| 28-33 | 14 (14.1) | 3 (4.8) |  |
| 34-36 | 43 (43.4) | 22 (34.9) |  |
| 37-41 | 39 (39.4) | 36 (57.1) |  |
| NA | 0 | 1 (1.6) |  |
| **Length of stay, days**  ***(Median [IQR])*** | 14.0  [8.0, 23.5] | 5.0  [4.0, 11.0] | <0.001 |
| **Birthweight, grams**  **(Median [IQR])** | 2450.0  [1890.0, 3260.0] | 2880.0  [2385.0, 3535.0] | 0.004 |
| **Birthweight** |  |  | 0.025 |
| <1000 g | 5 (5.1) | 1 (1.6) |  |
| 1000-1499 g | 8 (8.1) | 1 (1.6) |  |
| 1500-2499 g | 38 (38.4) | 16 (25.8) |  |
| ≥2500 g | 48 (48.5) | 44 (71.0) |  |
| **Weight at discharge, grams *(Median [IQR])*** | 2700.0  [2240.0, 3380.0] | 2940.0  [2436.0, 3460.0] | 0.262 |
| **Outborn** | 21 (21.2) | 10 (15.6) | 0.494 |
| **Apgar at 5 min <7** | 2 (2.0) | 2 (3.2) | 0.648 |
| **Ventilation at birth** | 21 (21.2) | 3 (4.7) | 0.003 |
| **Intubation** | 14 (14.1) | 3 (4.7) | 0.067 |
| **RDS** | 12 (12.1) | 2 (3.1) | 0.050 |
| **Any respiratory distress** | 45 (45.5) | 30 (46.9) | 0.987 |
| **Sepsis** | 11 (11.1) | 3 (4.7) | 0.252 |
| **Neurological event*** | 2 (2.0) | 1 (1.6) | 1.000 |
| **Major birth trauma**** | 1 (1.0) | 0 (0.0) | 1.000 |
| **Major malformation***** | 7 (7.1) | 2 (3.1) | 0.485 |

*Defined as convulsions, intraventricular haemorrhage, periventricular leukomalacia.

**Defined as fractured clavicle or humerus or fracture at any other site; brachial plexus paralysis and subgaleal hematoma.

***Defined for head and craniofacial structures: Anencephaly, encephalocele, holoprosencephaly, hydrocephaly, microphthalmia, anophthalmia, colobomas, microtia, cleft lip, cleft palate, severe micrognathia, macro and macroglossia. Neck: cystic hygroma. Chest: pectus excavatum, absent or hypoplastic clavicles. Back: meningomyelocele, spina bifida. Abdomen: omphalocele, gastroschisis. Genitalia: ambiguous genitalia. Extremities: absent or limb deficiencies, polydactyly, complete syndactyly, polysyndactyly, absent digits, ectrodactyly. Cardiovascular and great vessels: tetralogy of Fallot, truncus arteriosus, hypoplastic left heart, ventricular or atrial septal defect, transposition of the great vessels, interrupted aortic arch type B, total anomaly of pulmonary venous return, hypoplasia or coarctation of the aorta.

Abbreviations: IQR, interquartile range; RDS, respiratory distress syndrome.

**Supplementary Table 2.** Newborns’ characteristics during three COVID-19 pandemic periods.

|  | **Overall**  **n (%)** | **Pre-pandemic (T_0_)**  **n (%)** | **Low COVID-19 incidence (T_1_)**  **n (%)** | **High COVID-19 incidence (T_2_)**  **n (%)** | ***p-value*** |
| --- | --- | --- | --- | --- | --- |
| N | 99 | 40 | 39 | 20 |  |
| **Sex** | | | | | |
| Female | 54 (54.5) | 20 (50.0) | 18 (46.2) | 16 (80.0) | 0.034 |
| Male | 45 (45.5) | 20 (50.0) | 21 (53.8) | 4 (20.0) | 0.034 |
| **Gestational age, weeks** | | | | | |
| <=27 | 3 (3.0) | 1 (2.5) | 1 (2.6) | 1 (5.0) | 1.000 |
| 28-33 | 14 (14.1) | 6 (15.0) | 7 (17.9) | 1 (5.0) | 0.424 |
| 34-36 | 43 (43.4) | 20 (50.0) | 14 (35.9) | 9 (45.0) | 0.444 |
| 37-41 | 39 (39.4) | 13 (32.5) | 17 (43.6) | 9 (45.0) | 0.510 |
| **Unit of admission** | | | | | |
| NICU | 36 (36.4) | 6 (15.0) | 18 (46.2) | 12 (60.0) | 0.001 |
| Semi-intensive care | 63 (63.6) | 34 (85.0) | 21 (53.8) | 8 (40.0) | 0.001 |
| Length of stay, days  (Median [IQR]) | 14.0  [8.0, 23.5] | 15.0  [9.0, 28.5] | 14.0  [8.0, 23.5] | 9.0  [6.5, 18.8] | 0.327 |
| Birthweight, grams | 2450.0  [1890.0, 3260.0] | 2294.5  [1731.0, 3242.5] | 2320.0  [2057.5, 3333.5] | 2944.5  [2437.5, 3289.8] | 0.253 |
| **Birthweight** | | | | | |
| <1000 g | 5 (5.1) | 2 (5.0) | 2 (5.1) | 1 (5.0) | 1.000 |
| 1000-1499 g | 8 (8.1) | 4 (10.0) | 4 (10.3) | 0 (0.0) | 0.355 |
| 1500-2499 g | 38 (38.4) | 18 (45.0) | 15 (38.5) | 5 (25.0) | 0.339 |
| ≥2500 g | 48 (48.5) | 16 (40.0) | 18 (46.2) | 14 (70.0) | 0.084 |
| **Weight at discharge, grams**  **(Median [IQR])** | 2700.0  [2240.0, 3380.0] | 2465.0  [2100.0, 3290.0] | 2670.0  [2370.0, 3410.0] | 3120.0  [2685.0, 3462.5] | 0.083 |
| Outborn | 21 (21.2) | 8 (20.0) | 10 (25.6) | 3 (15.0) | 0.624 |
| Apgar at 5 min <7 | 2 (2.0) | 0 (0.0) | 1 (2.6) | 1 (5.0) | 0.686 |
| Ventilation at birth | 21 (21.2) | 10 (25.0) | 8 (20.5) | 3 (15.0) | 0.697 |
| Intubation | 14 (14.1) | 7 (17.5) | 4 (10.3) | 3 (15.0) | 0.654 |
| RDS | 12 (12.1) | 6 (15.0) | 5 (12.8) | 1 (5.0) | 0.664 |
| Any respiratory distress | 45 (45.5) | 21 (52.5) | 19 (48.7) | 5 (25.0) | 0.117 |
| Sepsis | 11 (11.1) | 7 (17.5) | 2 (5.1) | 2 (10.0) | 0.243 |
| Neurological event* | 2 (2.0) | 1 (2.5) | 1 (2.6) | 0 (0.0) | 1.000 |
| Major birth trauma** | 1 (1.0) | 1 (2.5) | 0 (0.0) | 0 (0.0) | 1.000 |
| Major malformation*** | 7 (7.1) | 4 (10.0) | 2 (5.1) | 1 (5.0) | 0.785 |

*Defined as convulsions, intraventricular haemorrhage, periventricular leukomalacia.

**Defined as fractured clavicle or humerus or fracture at any other site; brachial plexus paralysis and subgaleal hematoma.

***Defined for head and craniofacial structures: Anencephaly, encephalocele, holoprosencephaly, hydrocephaly, microphthalmia, anophthalmia, colobomas, microtia, cleft lip, cleft palate, severe micrognathia, macro and macroglossia. Neck: cystic hygroma. Chest: pectus excavatum, absent or hypoplastic clavicles. Back: meningomyelocele, spina bifida. Abdomen: omphalocele, gastroschisis. Genitalia: ambiguous genitalia. Extremities: absent or limb deficiencies, polydactyly, complete syndactyly, polysyndactyly, absent digits, ectrodactyly. Cardiovascular and great vessels: tetralogy of Fallot, truncus arteriosus, hypoplastic left heart, ventricular or atrial septal defect, transposition of the great vessels, interrupted aortic arch type B, total anomaly of pulmonary venous return, hypoplasia or coarctation of the aorta.

Abbreviations: IQR, interquartile range; RDS, respiratory distress syndrome.

**Supplementary Table 3.** Median (IQR) scores for each specific PSS:NICU (Parental Stressor Scale in the NICU) answer in three COVID-19 pandemic periods.

|  | **Overall** | **Pre-**  **pandemic (T_0_)** | **Low**  **COVID-19 incidence (T_1_)** | **High**  **COVID-19 incidence (T_2_)** | ***p-value*** | |
| --- | --- | --- | --- | --- | --- | --- |
| N | 152 | 62 | 56 | 34 |  | |
| Presence of monitors and equipment | 2.0 [1.0, 3.0] | 2.0 [1.0, 3.0] | 2.0 [1.0, 3.0] | 2.0 [2.0, 3.0] | 0.102 | |
| Constant noises of monitors and equipments | 2.0 [2.0, 3.0] | 2.0 [1.2, 3.0] | 2.0 [1.0, 3.0] | 2.0 [2.0, 3.0] | 0.954 | |
| Sudden noises of monitor alarms | 3.0 [2.0, 4.0] | 3.0 [2.0, 3.8] | 3.0 [2.0, 4.0] | 3.0 [2.0, 4.0] | 0.461 | |
| Other sick babies in the room | 1.0 [1.0, 2.0] | 1.0 [1.0, 2.0] | 1.0 [1.0, 2.0] | 1.0 [1.0, 2.8] | 0.313 | |
| Large number of nurses and doctors in NICU | 1.0 [1.0, 1.0] | 1.0 [1.0, 1.0] | 1.0 [1.0, 1.0] | 1.0 [1.0, 1.0] | 0.558 | |
| Having a ventilator to breathe for baby | 1.0 [0.0, 2.0] | 1.0 [0.0, 2.8] | 1.0 [0.0, 2.0] | 0.0 [0.0, 2.0] | 0.403 | |
| Tubes and equipments on or near my baby | 2.0 [1.0, 3.0] | 2.0 [1.2, 3.0] | 2.0 [1.0, 3.0] | 2.0 [1.0, 3.8] | 0.972 | |
| Bruises, cuts or incisions on my baby's body | 1.0 [0.0, 3.0] | 1.0 [0.0, 2.0] | 0.0 [0.0, 3.0] | 1.0 [0.0, 3.8] | 0.771 | |
| Unusual color of my baby | 1.0 [0.0, 3.0] | 1.0 [0.0, 2.0] | 1.0 [0.0, 2.2] | 0.0 [0.0, 3.0] | 0.891 | |
| Unusual or abnormal breathing patterns of my baby | 1.0 [0.0, 3.2] | 1.0 [0.0, 3.0] | 1.0 [0.0, 3.0] | 1.5 [0.0, 4.0] | 0.405 | |
| Small size of my baby | 1.0 [0.0, 2.0] | 2.0 [0.0, 3.0] | 0.5 [0.0, 2.0] | 1.0 [0.0, 2.0] | 0.072 | |
| Wrinkled appearance of my baby | 0.0 [0.0, 1.0] | 1.0 [0.0, 1.0] | 0.0 [0.0, 1.0] | 0.5 [0.0, 1.0] | 0.189 | |
| Seeing needles and tubes being put on my baby | 3.0 [1.0, 4.0] | 3.0 [2.0, 4.0] | 2.0 [0.0, 3.0] | 3.0 [1.0, 4.0] | 0.168 | |
| Baby fed by tube or intravenous line | 2.0 [0.0, 3.0] | 2.0 [2.0, 3.0] | 2.0 [0.0, 4.0] | 2.0 [0.0, 3.8] | 0.975 | |
| Seeing my baby in pain | 3.0 [0.0, 4.0] | 2.0 [0.0, 4.0] | 2.0 [0.0, 3.0] | 3.5 [0.2, 4.0] | 0.091 | |
| Seeing my baby to be sad | 1.0 [0.0, 3.0] | 2.0 [0.0, 3.0] | 0.0 [0.0, 3.0] | 1.0 [0.0, 4.0] | 0.554 |  |
| The limp or weak appearance of my baby | 1.0 [0.0, 2.2] | 2.0 [0.0, 2.8] | 0.0 [0.0, 2.0] | 1.0 [0.0, 3.0] | 0.171 |  |
| Jerky movements of my baby | 2.0 [0.8, 3.0] | 1.5 [0.2, 3.0] | 2.0 [0.0, 3.0] | 1.5 [1.0, 3.0] | 0.805 |  |
| Baby not crying like other babies | 0.0 [0.0, 0.2] | 0.0 [0.0, 0.0] | 0.0 [0.0, 1.0] | 0.0 [0.0, 0.0] | 0.925 |  |
| **Being separated from my baby *** | 4.0 [3.0, 5.0] | 4.0 [3.0, 5.0] | 4.0 [4.0, 5.0] | 5.0 [4.0, 5.0] | **0.008** |  |
| Not feeding my baby myself | 2.0 [1.0, 4.0] | 2.0 [1.0, 4.0] | 2.0 [0.8, 4.0] | 3.0 [1.0, 4.0] | 0.395 |  |
| Not being able to care for my baby myself | 2.0 [0.0, 3.0] | 2.0 [1.0, 3.0] | 2.0 [0.0, 3.0] | 2.5 [0.0, 4.0] | 0.281 |  |
| Not being able to hold my baby when I want | 3.0 [2.0, 4.0] | 3.0 [1.0, 4.0] | 3.0 [2.0, 4.0] | 4.0 [2.2, 5.0] | 0.234 |  |
| Feeling helpless and unable to protect my baby from painful procedures | 3.0 [1.0, 5.0] | 3.0 [1.0, 5.0] | 3.0 [0.8, 5.0] | 4.0 [2.0, 5.0] | 0.691 |  |
| Feeling helpless about how to help my baby during this time | 4.0 [2.0, 4.2] | 3.0 [1.2, 4.8] | 3.0 [2.0, 4.0] | 4.0 [3.0, 4.8] | 0.345 |  |
| Not having time to be alone with my baby | 2.0 [0.0, 3.0] | 2.0 [0.0, 3.0] | 2.0 [1.0, 3.0] | 3.0 [1.2, 3.8] | 0.134 |  |

**Supplementary Table 4.** Frequencies of IPP (Index of Parental Participation) positive answers in three COVID-19 pandemic periods.

|  | **Pre-pandemic**  **(T_0_)** | | **Low**  **COVID-19 incidence (T_1_)** | | **High**  **COVID-19 incidence (T_2_)** | | ***p-value*** |
| --- | --- | --- | --- | --- | --- | --- | --- |
|  | N=62 | | N=56 | | N=34 | |  |
| IPP question | Yes | % | Yes | % | Yes | % |  |
| ***Activities related to daily living*** | | | | | | | |
| Fed the baby with breast milk | 38 | 61.3 | 42 | 75.0 | 25 | 73.5 | 0.19 |
| Fed the baby with formula milk | 30 | 48.4 | 27 | 48.2 | 16 | 47.1 | 0.96 |
| **Changed clothes** | **26** | **41.9** | **35** | **62.5** | **21** | **61.8** | **0.048** |
| **Changes nappies** | **41** | **66.1** | **49** | **87.5** | **25** | **73.5** | **0.025** |
| Helped with cleaning | 46 | 74.2 | 39 | 69.6 | 24 | 70.6 | 0.849 |
| Settled for sleep or nap | 55 | 88.7 | 48 | 85.7 | 30 | 88.2 | 0.877 |
| ***Providing confort*** | | | | | | | |
| Comforted baby when upset | 50 | 80.6 | 54 | 96.4 | 29 | 85.3 | 0.066 |
| Comforted baby during a painful procedure | 20 | 32.3 | 14 | 25.0 | 13 | 38.2 | 0.381 |
| Spent quiet time interacting with baby | 57 | 91.9 | 54 | 96.4 | 33 | 97.1 | 0.435 |
| Stroked baby/rubbed back | 60 | 96.8 | 55 | 98.2 | 34 | 100.0 | 0.55 |
| Talked to the baby | 61 | 98.4 | 56 | 100.0 | 33 | 97.1 | 0.477 |
| Skin to skin contact with the baby for at least 1 hour | 32 | 51.6 | 29 | 51.8 | 15 | 44.1 | 0.738 |
| Any form of play | 17 | 27.4 | 21 | 37.5 | 9 | 26.5 | 0.365 |
| ***Advocating*** | | | | | | | |
| Asked physician for baby’s condition | 58 | 93.5 | 54 | 96.4 | 29 | 85.3 | 0.292 |
| Asked nurses for baby’s condition | 60 | 96.8 | 56 | 100.0 | 31 | 91.2 | 0.213 |
| Asked staff about provided baby's care | 60 | 96.8 | 48 | 85.7 | 29 | 85.3 | 0.138 |
| Asked staff to explain something that you did not understand | 42 | 67.7 | 39 | 69.6 | 29 | 85.3 | 0.087 |
| Told staff about something your baby needed | 43 | 69.4 | 30 | 53.6 | 21 | 61.8 | 0.253 |
| Told staff about what your baby like/dislike of the daily routine | 16 | 25.8 | 18 | 32.1 | 10 | 29.4 | 0.741 |
| Suggested to the staff a different way or time for doing something that you thought would be better for your baby | 9 | 14.5 | 11 | 19.6 | 9 | 26.5 | 0.321 |
| ***Technical task*** | | | | | | | |
| Helped the baby feeding with nasogatric tube or by cup | 35 | 56.5 | 33 | 58.9 | 21 | 61.8 | 0.734 |
| Changed position to the baby | 41 | 66.1 | 41 | 73.2 | 20 | 58.8 | 0.449 |
| Helped nurses to give oral medications | 14 | 22.6 | 8 | 14.3 | 5 | 14.7 | 0.451 |
| Helped nurse in doing other procedures | 29 | 46.8 | 16 | 28.6 | 12 | 35.3 | 0.123 |
| Kept track of the baby intake (feeding and fluids) and tell the nurse | 27 | 43.5 | 29 | 51.8 | 16 | 47.1 | 0.717 |
| Kept track of how often the baby urinates and tell the nurse | 19 | 30.6 | 18 | 32.1 | 13 | 38.2 | 0.7 |
| Kept track of how often the baby defecates and tell the nurse | 27 | 43.5 | 21 | 37.5 | 14 | 41.2 | 0.832 |
| Kept track if the baby had regurgitation/vomiting and tell the nurse | 26 | 41.9 | 19 | 33.9 | 19 | 55.9 | 0.092 |
| Helped detecting changes in color of your baby, respiratory patter, body temperature | 21 | 33.9 | 18 | 32.1 | 13 | 38.2 | 0.721 |
| Helped detecting changes tone, movements, reactions, sleep/awake cycles | 22 | 35.5 | 17 | 30.4 | 12 | 35.3 | 0.767 |
